# Clinical Report of a Case of Hepatic Abscess

**Published:** 1893-08

**Authors:** A. J. Bloch

**Affiliations:** New Orleans, La.; Visiting Physician to Charity Hospital; Clinical Assistant to the Chair of Physical Diagnosis and Practice of Medicine, New Orleans Polyclinic; Assistant Instructor in Physical Diagnosis, Tulane Medical College


### CLINICAL REPORT OF A CASE OF HEPATIC ABSCESS.*

By A. J. BLOCH, M. D.,
Visiting Physician to Charity Hospital; Clinical Assistant to the Chair of Physical Diagnosis and Practice of Medicine, New Orleans Polyclinic; Assistant Instructor in Physical Diagnosis, Tulane Medical College.

My only excuse to read a paper before you this evening is prompted by a desire to furnish you with my unfortunate experience, which, probably, might be of some benefit to some of our members.

I was called on the 30th of March last to see a gentleman sent

* Read before Orleans Parish Medical Society, April 29, 1893.

from the country by his physicians to see whether surgical treatment would not be of benefit to him. Mr. B. was a native of Louisiana; his family history was free from tuberculosis or syphilis. Prior to January, 1892, he enjoyed excellent health, at which time he was first confined to his bed by fever and pain in the right side. After prolonged medical treatment he experienced no relief, and came five months subsequently to New Orleans. This proving of slight benefit to him he returned home, and again came to New Orleans, this latter time five months ago. Being discouraged, having experienced no benefit, he determined to return home and die. Whilst on the train he was suddenly taken with most violent pains in the affected side, and in another moment he coughed up what he supposed to be a quantity of blood, but which subsequently proved to be liver pus.

The diagnosis of his trouble was quite evident now; his general condition being so much below par, his physician at home placed him on cod-liver oil, quinine and a cough mixture. They did not attempt a free incision to remove the septic material, not knowing exactly where to locate the pus cavity. Thus his condition remained, constantly coughing up pus, continuous septic fever and great progressive emaciation.

Upon taking charge of his case I immediately explored and located a pus cavity in the right lobe of the liver. I could not make out any definite destruction of lung tissue. There was an abundance of râles; these, however, might have been due to accumulated pus in the bronchial tubes. Dullness was well marked at the lower lobe of the right lung. It was quite evident to me that a chronic pneumonia must have supervened from a constant irritation of the septic material coughed up. The day following my first examination I had him sent to the New Orleans Sanitarium, and on April 1, under chloroform, I resected the rib and made a large opening into the abscess cavity, removing fully one pint of very foul pus.

Fearing to irrigate under chloroform, as the cavity communicated with the lung and the water might clog up the latter and produce death from asphyxia, I packed the cavity with iodoform gauze and had the patient removed to bed. The patient rallied well; his pulse, as before the operation, was 155 a minute; his respirations 40. The following day I irrigated with a 2 per cent. solution of carbolic acid and substituted a drainage tube for the gauze. The change in his condition the next week was phenomenal; his temperature became normal; his pulse was reduced to 110, stronger and fuller; his appetite was inordinate, eating or asking to be fed whenever awake; his respirations from 24 to 28 a minute. He had been taking a tonic of iron and quinine, and when constipated a little sulphate of magnesia was given. Each day I irrigated the cavity with the carbolized solution, after which I injected peroxide of hydrogen, followed by iodoform and glycerine (ʒiv to ℥vi). On April 15 I went to pay my daily visit. I found my patient in a very gratifying state; he complained a little of his cough, but so confident did I feel in a future recovery that I promised to let him sit up in ten days. He was prepared to be dressed by the nurse in attendance, the patient assisting himself in the preparation. As soon as I introduced the nozzle of the syringe into the wound he began to cough; this was nothing unusual, as I experienced the same difficulty every day. I stopped for a moment, which gave him relief, and began very gently to remove the pus. This time the cough became violent; my patient became cyanotic; I immediately grasped his pulse; it could not be felt; I tried every means possible to revive him, with no result; he was dead. I was unable to hold an autopsy, and can account for his death by shock only, produced by a rupture of liver attachments, with a flow of pus and water into the abdominal cavity, superinduced by the cough.

The points of interest in this case, gentlemen, must be very apparent to you. Death might have ensued from several factors; firstly, from asphyxia or drowning the patient by irrigation; secondly, from shock resulting from a rupture of adhesions between the liver, abdominal wall and diaphragm. Another cardinal point is the danger of delay in making an early incision to prevent a communication with the bronchial tube. Once this communication is formed the graver does the condition of our patient become. The constant passage of septic material through a lung, no matter how healthy, must leave destruction in its wake, and should we succeed in effecting a cure of the primary and graver trouble, we leave a suitable nidus for the propagation of the tubercle bacillus. I feel confident that my patient suffered from some such trouble.

The conclusion I draw from this case is: All suspected cases of liver abscesses should be explored early; a negative result will not increase the danger of the patient, a positive one will surely be of inestimable benefit. If pus is located an early incision should be made and the cavity thoroughly evacuated. If a communication has already formed with the bronchial tube irrigation should never be practiced, but in its place the cavity should be constantly packed with iodoform gauze. The latter will absorb the pus and disinfect the cavity, and the patient will be spared the danger from which my patient died.

For discussion see page 343.

